# A Hybrid Titanium-Softmaterial, High-Strength, Transparent Cranial Window for Transcranial Injection and Neuroimaging

**DOI:** 10.3390/bios12020129

**Published:** 2022-02-18

**Authors:** Nana Yang, Fengyu Liu, Xinyue Zhang, Chenni Chen, Zhiyuan Xia, Su Fu, Jiaxin Wang, Jingjing Xu, Shuang Cui, Yong Zhang, Ming Yi, You Wan, Qing Li, Shengyong Xu

**Affiliations:** 1 Key Laboratory for the Physics & Chemistry of Nanodevices, Department of Electronics, Peking University, Beijing 100871, China; yangnana@pku.edu.cn (N.Y.); xujj@sdu.edu.cn (J.X.); 2 Neuroscience Research Institute, Department of Neurobiology, School of Basic Medical Sciences, Peking University, Beijing 100191, China; ccn@pku.edu.cn (C.C.); sufu@bjmu.edu.cn (S.F.); jiaxinw@bjmu.edu.cn (J.W.); cshuang@bjmu.edu.cn (S.C.); yongzhang@hsc.pku.edu.cn (Y.Z.); mingyi@bjmu.edu.cn (M.Y.); ywan@hsc.pku.edu.cn (Y.W.); 3 Key Laboratory for Neuroscience, Ministry of Education/National Health Commission, Peking University, Beijing 100191, China; 4 Center of Digital Dentistry, Peking University School and Hospital of Stomatology, Beijing 100081, China; zhangxinyue@pkuss.bjmu.edu.cn (X.Z.); qingli@bjmu.edu.cn (Q.L.); 5 National Engineering Laboratory for Digital and Material Technology of Stomatology, Beijing 100081, China; 6 Department of Material Science and Engineering, College of Engineering, Peking University, Beijing 100871, China; xiazy@pku.edu.cn; 7 School of Microelectronics, Shandong University, Jinan 250100, China

**Keywords:** Ti-PDMS cranial window, high-strength, high-transparency, transcranial injection, 3D printing, two-photon imaging

## Abstract

A transparent and penetrable cranial window is essential for neuroimaging, transcranial injection and comprehensive understanding of cortical functions. For these applications, cranial windows made from glass coverslip, polydimethylsiloxane (PDMS), polymethylmethacrylate, crystal and silicone hydrogel have offered remarkable convenience. However, there is a lack of high-strength, high-transparency, penetrable cranial window with clinical application potential. We engineer high-strength hybrid Titanium-PDMS (Ti-PDMS) cranial windows, which allow large transparent area for *in vivo* two-photon imaging, and provide a soft window for transcranial injection. Laser scanning and 3D printing techniques are used to match the hybrid cranial window to different skull morphology. A multi-cycle degassing pouring process ensures a good combination of PDMS and Ti frame. Ti-PDMS cranial windows have a high fracture strength matching human skull bone, excellent light transmittance up to 94.4%, and refractive index close to biological tissue. Ti-PDMS cranial windows show excellent bio-compatibility during 21-week implantation in mice. Dye injection shows that the PDMS window has a “self-sealing” to keep liquid from leaking out. Two-photon imaging for brain tissues could be achieved up to 450 µm in z-depth. As a novel brain-computer-interface, this Ti-PDMS device offers an alternative choice for *in vivo* drug delivery, optical experiments, ultrasonic treatment and electrophysiology recording.

## 1. Introduction

Studies on brain functions face a natural barrier that the brain is tightly protected underneath the skull. Without surgeries on the cranium, technical developments of 3D visualization of functional magnetic resonance imaging (fMRI) [1,2,3,4], magnetoencephalography (MEG) [5], positron emission computed tomography (PET) [6] sequence images, electroencephalogram (EEG) [7,8], functional near–infrared spectroscopy (fNIRS) [9,10], other non-invasive optical-based approaches [11,12,13,14] and multi-modal technologies [15,16] have enabled obtaining structural, electrical and functional information of the brain at a large scale. For example, fMRI provides an excellent aid for the study of psychiatric disorders [3,4]. However, for dynamic observation of neuronal and synaptic activities at the single-cell level down to molecular scales, craniotomy experiments are frequently required for implantation of cortical and deep-brain electrodes or electrode arrays [17,18,19,20,21,22,23,24,25,26]. For two-photon experiments and fluorescence measurements, cranial surgery is also performed in installation of many novel brain-computer-interfaces (BCIs) [27,28,29,30,31,32,33]. For these applications, a transparent artificial cranial window with good mechanical strength may play an important role.

On the other hand, the technique of direct drug delivery into brain tissues has attracted much attention in recent years [32,34,35,36,37,38,39]. Han et al. reported that injecting drugs into the extracellular space of brain tissues might provide an efficient path to overcome the blood-brain barrier (BBB) in the treatment of stroke [40,41,42]. Trans-cranium injection of drugs may also benefit developing novel clinic therapies for brain tumor and other brain function disorders [38,43]. For trans-cranium injection, a penetrable soft-material cranial window would be a suitable choice. Thus, an ideal high-strength, high-transparency, penetrable and chronic cranial window is needed.

Recently, several techniques, such as thinning skull [29,44,45,46,47,48,49,50], transparent glass window [31,51,52,53,54] or polydimethylsiloxane (PDMS) [32,36,55] window have been developed for chronic optical access to the cortex for cellular resolution imaging. For trans-cranium operation or drug delivery, several approaches have been conducted [32,37,39,56] including a detachable glass coverslip cranial window [39]. However, each approach has its advantages and limitations.

The skull thinning technique, where a mouse skull is mechanically thinned to 15–30 µm, is biocompatible and allows long-term manipulations on a brain with normal states. At this thickness, the mouse cranium is almost transparent, allowing *in vivo* optical access [29]. For example, Han et al. performed *in vivo* two-photon imaging to investigate the dynamic changes in cerebral vessels and velocities of red blood cells (RBC) in mice following mild traumatic brain injury (mTBI) [46]. Without opening the cranium, this onsite thinning approach prevents infection and maintains the original status of the brain. However, a mechanically thinned skull is fragile so that access is restricted to a very small area, usually 0.1–0.3 mm^2^, therefore not suitable for large-scale observation [29,31,47,48]. Furthermore, a thinned skull may regrow 2–3 days after the operation, and re-thinning of the skull [27,29] necessary for repeated imaging increases surgery difficulty and measurement errors [57].

Another commonly applied technique for transparent cranial window is to replace part of the skull with a device made of transparent materials [32,58,59,60,61,62,63], such as glass coverslip [31,51,52,53,54], PDMS [32,36], Plexiglas [58], polyetheretherketone-silicone [59], silicone hydrogel [60], “See-Shell” [61] and curved glass (crystal skull) [62]. These materials are biocompatible, and allow visible light or other wavelengths to uniformly penetrate over a large area. *In vivo* two-photon imaging was used to examine the neural activities and microglia morphology in mice implanted with these transparent materials. Furthermore, several approaches have been constructed for trans-cranium injection. Goldey et al. designed a detachable glass coverslip cranial window for brain micro-injection [39]. Roome and Kuhn et al. designed a glass coverslip cranial window with a silicone plug and performed on-site injection of calcium-sensitive dye into brain cortex [37]. A similar method was used by Rossi et al. to study the neural activity associated with focal cortical seizures [50]. Takehara et al. proposed an improved window with integrated microfluidic channels for delivering chemicals or drugs into brain [56]. However, for large animals, glass and crystal are fragile, while materials such as PDMS are not rigid enough to protect the brain.

Ideally, a hybrid metal-softmaterial cranial window may have both high mechanical strength and excellent transparency. Titanium (Ti) mesh is a typical metallic cranioplasty material for its excellent biocompatibility, high strength and perfect structural stability [64,65,66,67,68,69,70]. Meanwhile, PDMS is a soft material with high transmittance, biocompatibility, and penetrability [71,72,73,74,75,76,77,78]. In this paper, we demonstrate a multifunctional cranial window device made from Ti and PDMS by a novel process. The hybrid Ti-PDMS device shows high mechanical strength similar to that of human cranium, excellent bio-compatibility and high-light transmittance, and allows high ratio of transparent area for *in vivo* observation and two-photon imaging. In addition, the soft-material part of the device is transparent and keep excellent elasticity for a long period that allow trans-window injection without leaking. Laser scanning and 3D printing techniques help to fabricate ideal shape and size to match the requirements for *in vivo* experiments on different size animals.

## 2. Materials and Methods

### 2.1. Design and Fabrication of Titanium Alloy Frames

Titanium (Ti) alloy frames with different shapes and sizes were designed to match various applications with 3Dmax software (Autodesk, San Rafael, CA, USA). Figure S1A–D in Supplementary Materials showed the design patterns of Ti frames suitable for mice. Figure S1A were square frames with grids defining varied inner opening shapes. Figure S1B showed a Ti frame with small locking parts. Figure S1C showed a Ti frame with small clamping parts. Figure S1D showed devices with both locking and clamping parts. In craniotomy operation, the locking parts were inserted into the gap between skull bone and brain tissue, and firmly fixed a Ti-PDMS cranial window to cranium without screws. The clamping parts were helpful for clamping the Ti-PDMS cranial window with tweezers.

Figure S1E,F in Supplementary Materials showed design patterns for rats with different grid pattern, small locking parts and clamping parts.

To demonstrate the feasibility for application in large animals, 7 kinds of 50 mm diameter circular Ti frames were designed. The thickness was kept constantly as 1.0 mm. The inner part of the frames was covered with hexagonal meshes with length was designed to be 2.0 mm, 2.5 mm, 3.0 mm, 3.5 mm, 4.0 mm, 5.0 mm, and 7.0 mm, respectively. Figure S1G in Supplementary Materials showed the designed pattern and sizes of one typical large Ti frame. The grid width of all these samples was kept 0.3 mm.

Ti frames were fabricated by a direct metal laser melting (DMLM) device (DMP Flex 350, 3D Systems, Rock Hill, SC, USA) in a standard procedure. The designs of Ti frames were exported into stereolithography (STL) format, and then transferred to a control software of 3D printer. The Ti frames were formed with alloy powders (Ti-6Al-4V, Ti Gr5, 3D Systems, Rock Hill, SC, USA, for specific ingredients, see Table S1 in Supplementary Materials) in a layer-by-layer manner and were sintered with a 500-watt fiber laser. The scanning path of the laser was the optimized, and the sintering temperature was set to 1500 °C.

Laser scanning technique was applied to obtain precise 3D profiling of the skulls of three mice and three rats as the references for designs of Ti-based cranial windows. Skull specimen was shown in Figure S2A in Supplementary Materials. The 3D morphology and size data of this skull specimen was obtained by a laser scanner (Planmeca, Planmeca oy, Helsinki, Finland), as shown in Figure S2B in Supplementary Materials. Subsequently, 3D model of the rat skull specimen was exported into a STL format. The 3D model of the skull was modified to generate a circular location with a diameter of 8 mm at the position of the dorsal cerebral cortex via a 3D Sprint software (3D Systems, Rock Hill, SC, USA), as shown in Figure S2C in Supplementary Materials. Meanwhile, a matching Ti frame model was designed according to the morphology and size of the location, as shown in Figure S2D in Supplementary Materials.

The 3D model of a rat skull was fabricated with a different 3D printer (ProJet 2500 Plus, 3D Systems, Rock Hill, SC, USA) from liquid photosensitive resin solidified with a ultraviolet light source. After the printing procedure was completed, the wax support on the surface was removed by constant temperature ultrasonic cleaning.

### 2.2. Fabrication of the Hybrid Ti-PDMS Transparent Cranial Windows

The inner surface of as-fabricated Ti frames was mechanically polished to prevent damages to the brain tissue. After polishing, Ti frames were ultrasonically cleaned with deionized water, acetone, ethanol, and deionized water for 15 min and then dried with a nitrogen gun. Subsequently, Ti frames were placed in a convection oven incubator for 3 h at 80 °C to fully dry.

**Figure 1:**
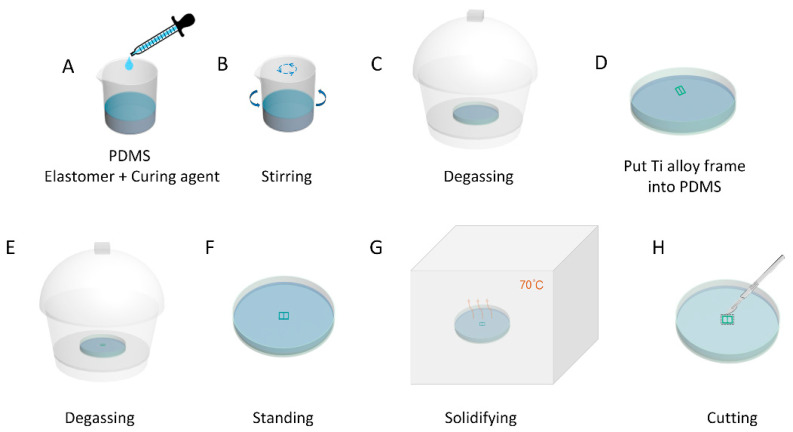
Schematic representation of the fabrication processes of a Ti-PDMS transparent cranial window device. (**A**) The PDMS base elastomer and curing agent were mixed in a ratio of 10:1. (**B**) The PDMS solution was stirred and degassed in a mixer. (**C**) The PDMS solution contained in the sterile cell culture dish was degassed in a vacuum desiccator. (**D**) Fully dried Ti frame was slowly put into the PDMS solution and completely immersed. (**E**) The PDMS solution containing the Ti frame in the sterile cell culture dish was degassed again in a vacuum desiccator. (**F**) The PDMS solution containing the Ti frame in the sterile cell culture dish was placed on the optical platform to stand still. (**G**) The PDMS solution containing the Ti frame in the sterile cell culture dish was solidified in a convection oven incubator. (**H**) Ti-PDMS cranial window device was disconnected from the PDMS matrix along the outer edge with a scalpel.

Ti-PDMS cranial window devices were prepared by filling PDMS (Sylgard 184, Dow Corning, Midland, MI, USA) windows in polished Ti frames with a home-made multi-cycle degassing pouring process, as show in Figure 1. First, the PDMS base elastomer and curing agent were mixed in a ratio of 10:1 (Figure 1A). The mixture was stirred (Figure 1B) and degassed in a mixer (AR-100, THINKY, Tokyo, Japan). An appropriate volume of the degassed PDMS solution was poured into a sterile cell culture dish and degassed in a vacuum desiccator for 30 min (Figure 1C). Next, fully dried Ti frames were slowly and completely immersed in the PDMS solution (Figure 1D). The entire sterile cell culture dish was placed in a vacuum desiccator again for second degassing process (Figure 1E). The entire sterile cell culture dish was placed on the optical platform and allowed to keep still for 2 h to ensure a uniform liquid thickness (Figure 1F). The entire sterile cell culture dish was transferred to a convection oven incubator for 4 h at 70 °C for solidifying (Figure 1G). The base of the oven should be flat when incubating the PDMS solution. Finally, the Ti-PDMS cranial window device was disconnected from the PDMS matrix along the outer edge with a scalpel (Figure 1H). By these processes, PDMS windows in uniform thickness without air-bubbles were obtained.

### 2.3. Characterization of Hybrid Ti-PDMS Cranial Windows

#### 2.3.1. Morphology and Hydrophobic Characterization

Photographs for the samples were taken with a digital camera (EOS800D, Canon, Tokyo, Japan). Micrographs of the surface morphology of Ti frames were obtained before and after polishing process with a scanning electron microscope (SEM, Quanta 600FEG, FEI, Brno, Czech Republic).

Surface roughness of the polished inner surface of Ti frames and Ti-PDMS cranial window samples was analyzed with an atomic force microscope (AFM, Dimension Icon, Bruker, Billerica, MA, USA). For each sample, five locations with area of 5 μm × 5 μm were chosen for measurement, and the average roughness (*R_a_*) was calculated from the results, as shown in Figure S3 in Supplementary Materials.

For evaluation of transparency in visible lights, the Ti-PDMS cranial windows were placed on a four-inch silicon chip with micro-patterns. Photographs of the patterns were taken through the PDMS window with a CCD (MC–D500U(C)/TP, Phenix, Shangrao, China) on the stereo-zoom optical microscope (Phenix, Shangrao, China).

The hydrophobicity of the PDMS film was measured by a contact angle detector (SDC-200SH, SINDIN, Dongguan, China) under ambient conditions. A drop of deionized water with volume of 3 μL was dropped on the surface of the PDMS film by a syringe. Immediately, the magnified image of the droplet was captured by a side camera, and the contact angle was measured by a software carried by the contact angle detector. To reduce the measurement error, five different positions of each sample was measured and the average contact angles was determined as the final result (Figure S4, in Supplementary Materials).

The weights of various Ti-PDMS cranial window samples were measured with an electronic scale (JJ124BC, G&G, Changshu, China).

#### 2.3.2. Mechanical Characterization

An electronic universal machine (Instron 5843, Canton, MA, USA) was used to characterize the mechanical properties of the Ti frames and Ti-PDMS cranial windows with diameter of 50.0 mm, thickness of 1.0 mm, and hexagonal grid mesh of different sizes. The testing device was composed of a pressure plate, a bottom bracket and a 1000 N load cell. To measure the fracture load, the pressure plate connected to the load cell applied a vertical stress at the center of the outer surface of each Ti-PDMS cranial window with a controlled velocity of 2.0 mm min^−1^ until the sample fractured. In this process, the load-displacement curve was automatically recorded. The fracture load was calculated from the recorded load-displacement data.

#### 2.3.3. Optical Characterization

The light transmittances of PDMS films and #1 glass coverslip were measured with an ultraviolet-visible-near-infrared (UV-Vis-NIR) spectrophotometer (Cary 5000, Varian, Palo Alto, CA, USA) in the wavelength range of 250–1500 nm. Five sets of PDMS sheets were measured. Each set consisted of 11 samples with thicknesses of 0.3 mm, 0.5 mm, 0.75 mm, 1.0 mm, 2.0 mm, 3.5 mm, 4.0 mm, 4.5 mm, 6.0 mm, 7.0 mm and 10.0 mm, respectively. The sheet size was kept constant as 2.0 cm × 2.0 cm. The #1 glass coverslips were commercially purchased (10212432C, LABSEE, Shanghai, China), they were 24 mm × 32 mm in size and 0.16 mm in thickness.

The refractive index of PDMS samples was characterized with a spectroscopic ellipsometer (UVISEL, HORIBA, Chilly-Mazarin, France). PDMS liquid was spin-coated (8000 rpm, 120 s) on a 4-inch silicon substrate to form a thin film with a thickness of 9.0 μm. After being solidified, the film was measured at five different positions for its refractive index in the wavelength range of 300–830 nm, and the average value was taken as the result.

### 2.4. Animals and Housing

Male and female C57BL/6 mice (20 to 30 g at the start of experiments) and Sprague-Dawley rats (230–250 g at the start of experiments) were provided by the Department of Laboratory Animal sciences, Peking University Health Science Center. All animals were housed under pathogen-free circumstances, with a 12 h alternating light/dark cycle and food and water available ad libitum. All animal experiments were approved by the Animal Care and Use Committee of Peking University Health Science Center (LA2017245, approved on 30 August 2017) and were performed in accordance with the Animal Management Rules of Ministry of Health of the People’s Republic of China. In these experiments, rats and mice were used for skull profiling. Mice were used for *in vivo* Ti-PDMS cranial window implantation.

### 2.5. Histological Staining

Mice were fully anesthetized in 1% pentobarbital sodium and transcardially perfused with phosphate-buffered saline (PBS) followed by 4% paraformaldehyde (PFA). The brains were extracted and stored overnight in 4% PFA for fixation. Then, the brain was kept in 20% sucrose followed by 30% sucrose overnight for dehydration. The brain was sectioned into 30 µm slices using a cryostat microtome (model 1950, Leica, Nussloch, Germany), and the slices were then mounted onto slides. Slices blocked with a buffer containing 5% bull serum albumin and 0.3% Triton X-100 for 1 h, and incubated with primary antibodies at 4 °C for 24 h. The primary antibodies used were as follows: goat anti-Iba-1 (1:500, Abcam, Cambridge, UK) and mouse anti-NeuN (1:500, Abcam, Cambridge, UK). Sections were then washed in PBS and incubated with secondary antibodies at room temperature for 90 min. The secondary antibodies used were as follows: Alexa Fluor 488-conjugated donkey anti-goat IgG (1:500, Abcam, Cambridge, UK), Alexa Fluor 594-conjugated donkey anti-mouse IgG (1:500, Abcam, Cambridge, UK). Then tissues were rinsed three times with PBS and DAPI (1:1000 dilution, Cell Signaling Technology, Danvers, MA, USA) was added to the last wash with PBS buffer for nuclear staining [79,80,81,82,83,84,85]. Next, the slices were mounted on glass slides, and covered with PDMS or #1 glass coverslips. Slices were imaged under an upright microscope (TCS-SP8 DIVE, Leica Microsystems, Wetzlar, Germany).

### 2.6. Ti-PDMS Cranial Window Implantation in Mice

The procedure for implantation of the Ti-PDMS cranial window was adapted from previously reported chronic glass window implantation protocols [28,31]. Mice (C57BL/6) were anesthetized in an induction chamber with 1–3% isoflurane (RWD Life Science, China). Their scalps were shaved and cleaned using standard aseptic surgical procedures, then their heads were firmly fixed using ear bars and a nose cone in the stereotaxic instrument (RWD, Shenzhen, China), as shown in Figure S5A in Supplementary Materials. The animals’ body temperatures were maintained at 36.5–37.5 °C throughout the procedure using a heating pad connected to a controller (DC temperature controller, RWD Life Science, Shenzhen, China), and their eyes were covered with eye protecting gel (Beijing Twinluck Pharmaceutical Co. Ltd., Bejing, China). Anesthetic depth was assessed every 15 min via toe pinch stimulus and anesthesia dosage adjusted as needed. A local anesthetic (1% lidocaine) was administered subcutaneously at the incision sites. The scalp was then carefully removed to expose the skull covering the dorsal cortice (Figure S5B, in Supplementary Materials). The remaining epidermis and debris were removed with 0.9% saline liquid swabs. The craniotomy (1 mm above bregma and 4 mm below bregma with a width of 5 mm) was calculated according to the size of Ti-PDMS and the desired brain area (Figure S5C, in Supplementary Materials). Afterwards, we carefully cut the skull along the outer edge of the titanium alloy frame (size of 5 mm × 5 mm × 0.35 mm) using a scalpel. The skull was removed using surgical forceps. The dura was intact over the entire exposed brain (Figure S5D, in Supplementary Materials). The prepared Ti-PDMS composite device was sterilized by soaking in 70% ethanol for 2–3 min followed by rinsing in sterile saline. Subsequently, the Ti-PDMS composite device (size = 5 mm × 5 mm, thickness = about 0.35 mm) was gently applied to fully cover the exposed brain tissue and aligned to the craniotomy. Immediately, dental resin (Super Bond C&B, Sun Medical, Japan) was applied along the edges of the Ti-PDMS device to cement it to the skull surface (Figure S5E,F, in Supplementary Materials). In order to protect the implant and underlying brain from light and physical impacts, two layers of heat release tape was fixed on the skull with cyanoacrylate glue to cover the Ti-PDMS cranial window. After implantation, mice were allowed to recover on a heating pad until ambulatory and then returned to a clean home cage. The antibiotics sulfamethoxazole (1 mg mL^−1^) and trimethoprim (0.2 mg mL^−1^) were chronically administered in the drinking water, and the animals were housed individually after surgery.

Before each imaging session, 70% ethanol or distilled water was used for Ti-PDMS cranial window cleaning. So far, we have conducted Ti-PDMS cranial window surgeries on 22 mice. The mice were placed in their home cages and their behavior was recorded with a camera for 30 min using a video tracking system (HDSPCAM, Shanghai, China).

### 2.7. Ti-PDMS Cranial Window Penetration, Dye Injection and Imaging

Glass micropipettes were used for dye or drug injection via the Ti-PDMS cranial window. The glass micropipette (~50 µm outer diameter, 4–6 MΩ resistance) were prepared for microinjection refered to previous study [86]. Precleaned glass tubes were drawn on a micropipette puller (PP-830, Narishige, Tokyo, Japan). In order to microinject into the brain, the length of the thin portion should be not less than 10 mm to minimize damage to brain tissue with a larger diameter segment of the tube. The tip of a micropipette was broken to an outer diameter of ~50 µm. The resistance was measured using patch clamp method. Micropipette resistances in the bath solution were 4–6 MΩ when the pipettes were filled with intracellular solution. Intracellular solution contained 114 mM K-gluconate, 6 mM KCl, 10 mM HEPES, 5 mM EGTA, 4 mM ATP-Mg and 0.3 mM GTP (pH was adjusted to 7.3 with KOH). The bath solution contained (mM) 140 NaCl, 2.5 KCl, 1 MgSO_4_, 1.3 CaCl_2_, 1.2 NaH_2_PO_4_, 10 glucose and 10 HEPES–NaOH, pH 7.4.

Before the injection operation, the mice (10 weeks after implantation surgery) were fully anesthetized with 2% isoflurane and then their heads were firmly secured to a stereotaxic instrument (RWD Life Science, Shenzhen, China). The micro-injector equipped with micropipette was assembled on the manipulator, and then the glass micropipette slid into the injection site after penetrating the Ti-PDMS cranial window under the control of the 3-axis micromanipulator of stereotaxic instrument [87,88]. The tip for injecting was positioned at 0.7 mm below the cortex surface.

We first tried single point injection. Hoechst 33258 (10 µg mL^−1^, 0.2 µL, 0.1 µL min^−1^, Merck, Burlington, MA, USA) was injected into the brain under the control of the microinjection control device. The glass micropipette was removed from the brain 5 min after the fluorescent dye injection. Two hours after microinjection, Hoechst in the injection site was imaged under an upright two-photon laser-scanning microscope (TCS-SP8 DIVE, Leica Microsystems, Wetzlar, Germany). Hoechst 33258 (magenta) was excited at 760 nm with 20–50 mW laser power measured after the objective (Figure S6, in Supplementary Materials).

In order to mark the surface of PDMS and brain, the reflected light of PDMS surface and brain surface were obtained with Leica TCS-SP8 DIVE microscope with excitation wavelength of 552 nm. The wavelength range of the detector was 548–558 nm. So, the blue color was due to the reflection from the surface of PDMS and brain (Figure S6). These methods refered to the previous study [89]. Image stacks were acquired at 512 × 512 pixels with a voxel size of 1.44 μm in x and y and a z-step of 3 μm.

As for testing the “self-sealing” property of PDMS, multiple injections at a single site were performed *in vitro*. In this experiment, we injected the diluted blue ink (Pilot, Isesaki, Japan) into a conical centrifuge tube (15 mL, Corning, Shanghai, China) filled with 0.9% saline by penetrating a PDMS membrane (thickness of 0.3 mm) with a commercial 1 mL syringe (Yuekang, Changzhou, China). After 15 repeated injections, the conical centrifuge tube was inverted and shaken, and then we observed whether there was fluid leakage from the injection site on the PDMS membrane (Figure S7, Video S1, in Supplementary Materials).

Next, we tried three point injections. DiI (0.1% in methanol, 0.2 µL, 0.1 µL min^−1^, Life Technologies, Carlsbad, CA, USA) was injected in first two points. CTB488 (1 mg mL^−1^ in PBS, 0.2 µL, 0.1 µL min^−1^, Life Technologies, Carlsbad, CA, USA) was injected in the third point. Two days after microinjection, DiI and CTB488 in injection sites were imaged under an upright laser-scanning microscope (TCS-SP8 DIVE, Leica Microsystems, Wetzlar, Germany).

## 3. Results and Discussions

### 3.1. 3D Printed Ti Supporting Frames with Different Size and Shape

To match the requirements for different applications, we used computerized 3D printing techniques to fabricate the titanium (Ti) alloy (Ti6Al4V) supporting frames, which served as the “bone” of the Ti-PDMS hybrid cranial window devices. The outside boundary shape was designed to match the location of skull, and the open windows within the boundary were confined with thin grids with hexagonal, triangular or rectangular shapes to match specific applications. For different animals with varied dimension of skull, we fabricated Ti frames from a small size of 5.0 mm for mice up to a diameter of 50.0 mm for potential applications in large animals such as porcine and primates.

**Figure 2:**
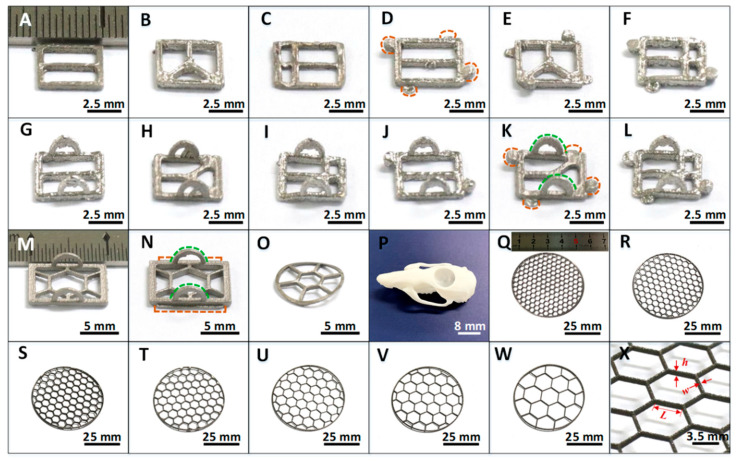
Optical images of the 3D printed Ti cranial frames with different shapes and sizes. (**A**–**L**) Small rectangular frames for mouse experiments. The orange dotted lines in (**D**,**K**) marked the locking parts on Ti frames, and the green dotted lines in (**K**) marked the clamping parts. (**M**,**N**) Rectangular frames for rat experiments. The orange and green dotted lines in (**N**), respectively, marked the locking and clamping parts on Ti frames. (**O**) A circular, curved frame for rat skull. (**P**) Photograph of a resin rat skull mold. (**Q**–**W**) 50 mm diameter circular Ti frames with hexagonal grid shape of different mesh sizes. (**X**) A close look at the Ti frame shown in (**T**), with grid length *L* of 3.5 mm, grid width *w* of 0.3 mm and the device thickness *h* of 1.0 mm.

Figure 2A–L showed 12 kinds of Ti frames with a rectangular shape of 5.0 mm × 5.0 mm, and a thickness of 0.3 mm. These 12 small frames were suitable for implantation in mice. Figure 2M,N presented larger frames with rectangular size of 10.0 mm × 8.0 mm and a thickness of 0.8 mm for rat application. Figure 2O was also a Ti frame for rat implantation, but it was fabricated with a curved shape to match a special location of the rat skull, whose exact shape was presented in Figure 2P. To obtain the precise shape of skull for certain animals, we used laser scanning techniques (see Materials and Methods, Figure S2 in Supplementary Materials) to reproduce the 3D morphology of a specific rat skull.

Figure 2Q–W showed 7 kinds of circular shape Ti frames with a diameter of 50.0 mm and hexagonal grid shape. These frames had a thickness of 1.0 mm. Figure 2X showed a part of a titanium alloy frame, in which the grid length (*L*) and width (*w*) of the hexagonal open windows and grid thickness (*h*) are highlighted with red arrows. For Figure 2Q–W, *L* values were 2.0 mm, 2.5 mm, 3.0 mm, 3.5 mm, 4.0 mm, 5.0 mm and 7.0 mm, respectively. Meanwhile, *w* kept the same, 0.3 mm. The device thickness *h* was 1.0 mm.

In addition, we designed various small locking or clamping parts on the Ti frames, as typically shown in Figure 2D,K,N, so that they were easily clamped with tweezers in implantation operation, and were firmly fixed on opened living animal skull without screws. By these designs, we thus provided a universal and flexible approach for *in vivo* studies of animals with different status and sizes.

### 3.2. Morphology and Transparency of Hybrid Ti-PDMS Cranial Window

**Figure 3:**
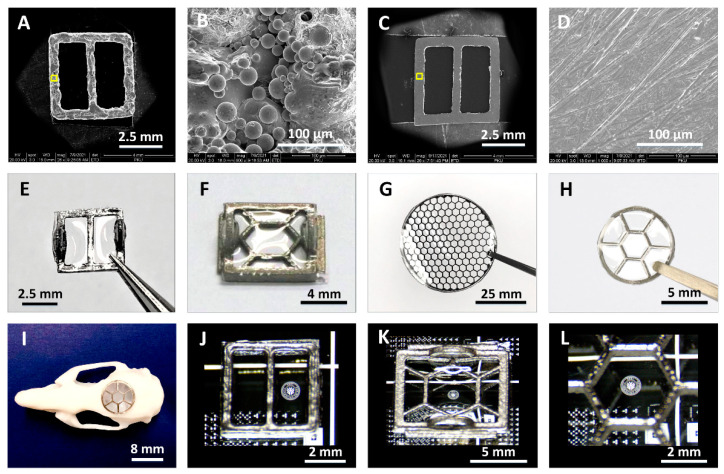
Images showing morphology and transparency of the Ti-PDMS cranial windows. (**A**,**B**) SEM images of the inner surface of as-fabricated Ti frame at different magnifications at (**A**) low and (**B**) high resolution. (**C**,**D**) SEM images of the sample after mechanical polishing process at different magnifications at (**C**) low and (**D**) high resolution. (**E**–**H**) Photographs of as-fabricated Ti-PDMS cranial windows, where a PDMS layer was mounted onto each polished Ti-frame. (**I**) Photograph of resin rat skull mold equipped with a curved Ti-PDMS cranial window. (**J**–**L**) Microscope images of the cranial windows in Figure (**E**–**G**), respectively, when they were placed on a silicon chip with a logo patterns.

For implantation in living animals, the inner surface of the Ti frames needs to be free of burrs to avoid damaging brain tissue. As shown in Figure 3A,B, the as-fabricated Ti frames in 3D printing process had a rough surface. Figure 3C,D presented scanning electron microscope (SEM) images of the polished surface of a Ti frame at varied scales. After mechanical polishing process, the average surface roughness (*Ra*) of Ti frames was remarkably reduced, which was measured to be 35.5 ± 5.3 nm over a measurement area of 5 μm × 5 μm (Figure S3A, in Supplementary Materials).

Our Ti-PDMS cranial window devices were fabricated by fixing PDMS window on the polished Ti frames via a special pouring process (see Materials and Methods, Figure 1). Figure 3E–G showed several typical Ti-PDMS cranial windows prepared for implantation experiments in mice, rats and large animals, respectively. Figure 3H showed a sample with curved shape. Figure 3I was a photograph showing that a curved Ti-PDMS sample installed on a resin rat skull mold. The unique Ti-PDMS process ensured cranial window samples were clear and free of gas bubbles. In Figure 3J–L, the details of the logo patterns underlying the Ti-PDMS cranial window were clearly visible. No bubbles were observed in the window region. These results prove that our fabricating processes for the Ti-PDMS windows are highly effective.

Compared with Ti frames, the surface roughness of the inner surface of Ti-PDMS samples was remarkably reduced. AFM measurements showed that the average roughness was only 5.1 ± 0.7 nm (Figure S3B, in Supplementary Materials), which was improved by 7 times. So, flatness of the hybrid Ti-PDMS cranial window had obviously improved. The exact weights of our Ti-PDMS cranial windows were measured to be 43.00 ± 6.69 mg, 0.21 ± 0.02 g, and 6.54 ± 0.53 g (Table S2, in Supplementary Materials), respectively, for mouse, rat and large animal as shown in Figure 2. The light weight makes them suitable for implantation in living animals.

### 3.3. Proportion of Transparent Area and Mechanical Properties of Ti-PDMS Device

**Figure 4:**
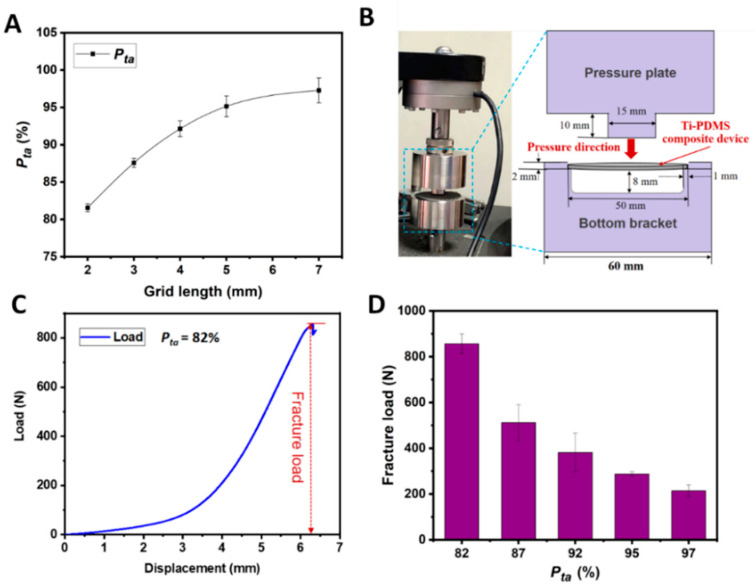
The proportion of transparent area (*P_ta_*) and mechanical property of a series Ti-PDMS cranial windows with diameter of 50.0 mm, grid width of 0.3 mm and device thickness of 1.0 mm. (**A**) *P_ta_* versus grid length of the Ti frames. Error bars indicated SD. (**B**) The electronic universal machine (left) and the schematic diagram for testing procedure (right). (**C**) A measured displacement-load curve taken from a sample with grid length of 2.0 mm and *P_ta_* 82%. The red dotted arrow indicates the fracture load. (**D**) The fracture load of Ti-PDMS cranial window samples versus the proportion of transparent area *P_ta_*. Error bars indicated SD.

The mechanical strength of the artificial Ti-PDMS cranial window plays a very important role in protecting the brain from injury. A given device thickness of the Ti supporting frame, this strength is directly related to the proportion of transparent area *P_ta_*, defined as *P_ta_* = *S_ta_*/*S_T_*, where *S_ta_* is the transparent area (i.e., the sum of PDMS window areas) and *S_T_* is the total area of the device. Figure 4A showed the *P_ta_* of a series of circular Ti frames with a constant 50.0 mm diameter and varied hexagonal meshes. For the grid length *L* of individual mesh of 2.0 mm, 3.0 mm, 4.0 mm, 5.0 mm and 7.0 mm, the *P_ta_* values were about 82%, 87%, 92%, 95%, and 97%, respectively. The high ratio of transparent area is beneficial for large-area observation of brain tissues. 

The mechanical strength of the Ti-PDMS cranial window device, in terms of fracture load, was measured with a standard procedure on an electronic mechanical testing instrument (see Materials and Methods). Figure 4B showed the photograph of the instrument and the way of loading test for the sample. Figure 4C showed a typical displacement-load curve of a Ti- PDMS cranial window with diameter of 50.0 mm, grid length of 2.0 mm, grid width of 0.3 mm, device thickness of 1.0 mm and a transparency portion *P_ta_* of 82%.

The load was gradually added on the Ti-PDMS device perpendicular to the device surface. Increase of the load first caused displacement of the center. Exceeding a certain threshold, part of the grids started to break, and the tested curve dropped sharply. The fracture load was defined as the load just before the device broke. As shown in Figure 4D, the fracture load of the series of Ti-PDMS cranial windows with *P_ta_* of 82%, 87%, 92%, 95% and 97% (Figure 4A) were 856.43 ± 42.67 N, 511.62 ± 77.90 N, 381.06 ± 84.72 N, 287.36 ± 9.78 N and 214.42 ± 25.18 N, respectively. These measured data were in the same range for those taken from human skull samples (184.6 N–940.1 N) [90].

### 3.4. Optical Properties of Transparent Ti-PDMS Cranial Window

**Figure 5:**
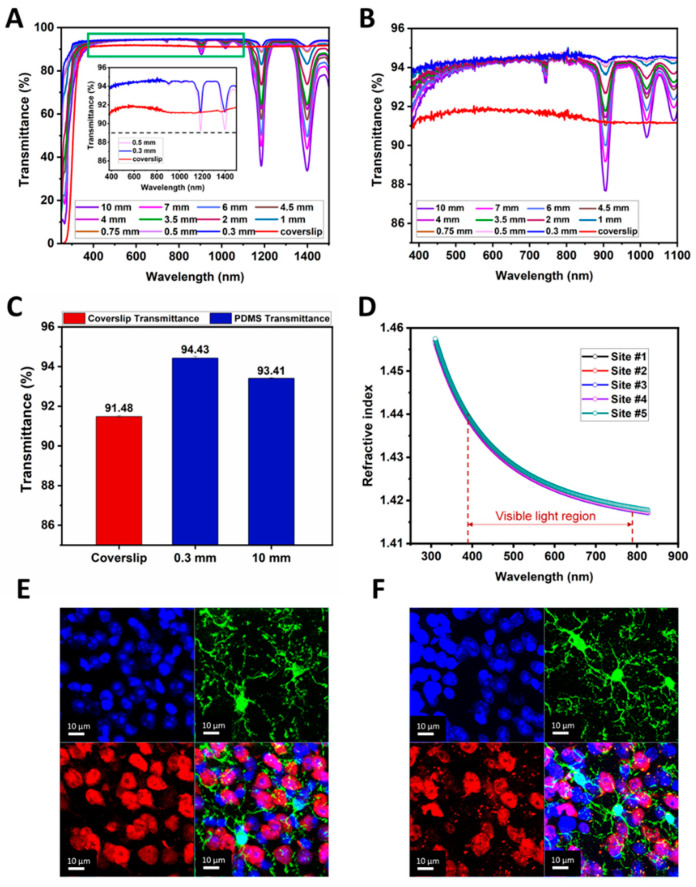
Optical properties of transparent Ti-PDMS cranial window. (**A**) A set of light transmittance-wavelength curves taken from 11 PDMS (thickness from 0.3 mm to 10.0 mm) and a #1 glass coverslip in the wavelength range of 250–1500 nm. Inset image showed the light transmittance of coverslip and PDMS with thickness of 0.3 mm and 0.5 mm in the wavelength range of 380–1500 nm. (**B**) An enlarged curves from (**A**) in the wavelength range of 380–1100 nm. (**C**) The average light transmittance in the wavelength range of 380–1100 nm for 2 PDMS sheets and #1 glass coverslip. Error bars indicated SD. (**D**) Refractive index data measured from 5 sites of a 4-inch diameter PDMS film in the wavelength range of 300–830 nm. (**E**) The high-resolution images of the fluorescent signal through PDMS. (**F**) The high-resolution images of the fluorescent signal through a glass coverslip.

PDMS is chosen as the light-transmitting element of Ti-PDMS cranial window, because it is a flexible and biocompatible polymer with excellent optical properties [71,72,73,74,75]. To demonstrate the optical property of the PDMS windows mounted on the Ti frames, we measured two key optical factors: light transmittance and refractive index. Figure 5A showed light transmittance in the wavelength range of 250–1500 nm for a set of 11 PDMS sheet samples (see Materials and Methods) with thickness from 0.3 mm to 10.0 mm, together with a #1 glass coverslip (thickness of 160 µm). The measurements were performed on an ultraviolet-visible-near-infrared (UV-Vis-NIR) spectrophotometer. The #1 glass coverslip was used as the “gold standard”, as such coverslips were widely applied in a variety of microscopic imaging experiments [61].

Figure 5B was an enlarged part of Figure 5A in the visible wavelength range (380–1100 nm). In this region, as the wavelength increases, the light transmittance of the PDMS film slowly rose. For PDMS sheets thinner than 10.0 mm, their transmittance was higher than that of #1 glass coverslip. Figure 5C presented the average light transmittances of a bunch of samples for the #1 glass coverslip and PDMS sheets with thicknesses of 0.3 mm and 10.0 mm in wavelength of 380–1100 nm. The light transmittance of PDMS films decreased as the thickness increases. The transmittance of a PDMS sheet even as thick as 10.0 mm (about 93.41%) was better than that of #1 glass coverslip (about 91.48%). Actually, the thickness of the cranial window currently used for imaging in monkeys was about 0.45 mm [59], the average transmittance of PDMS sheets thinner than 0.5 mm was above 93.95% in the wavelength range of 380–1500 nm, and the minimum transmittance was above 89%, as shown in the inset in Figure 5A. Clearly, Ti-PDMS cranial window is a better choice for *in vivo* measurements on living animals.

The refractive index was measured on five sites of a PDMS sheet spin-coated on a four-inch silicon substrate, as shown in Figure 5D. In visible light region (380–780 nm), the measured data decreased slightly as the wavelength increased. Compared with that of glass coverslip (~1.52) [91], the range of refractive index (1.42–1.44) was closer to the refractive index of biological tissue (1.38–1.41) [92]. So, in the *in vivo* optical experiments, Ti-PDMS cranial window has less image distortion.

In addition, the resolution of the fluorescent signal through PDMS and glass coverslip was compared in brain slice (30 µm, Figure 5E,F, Video S2 and S3 in Supplementary Materials). Under the same imaging parameters, the images of microglia with green fluorescence, the nucleus of neurons with red fluorescence, and the nuclei of all cells with blue fluorescence were all clearly visible through PDMS film and #1 cover glass. No significant difference was observed between PDMS and the glass coverslip, which was widely used in various long-term high-resolution imaging. Similar results have been reported in previously [32]. Therefore, we can infer that the PDMS window in the Ti-PDMS cranial window will not induce changes in fluorescence intensity during imaging.

### 3.5. Implantation and Functional Evaluation of Chronic Ti-PDMS Cranial Window

**Figure 6:**
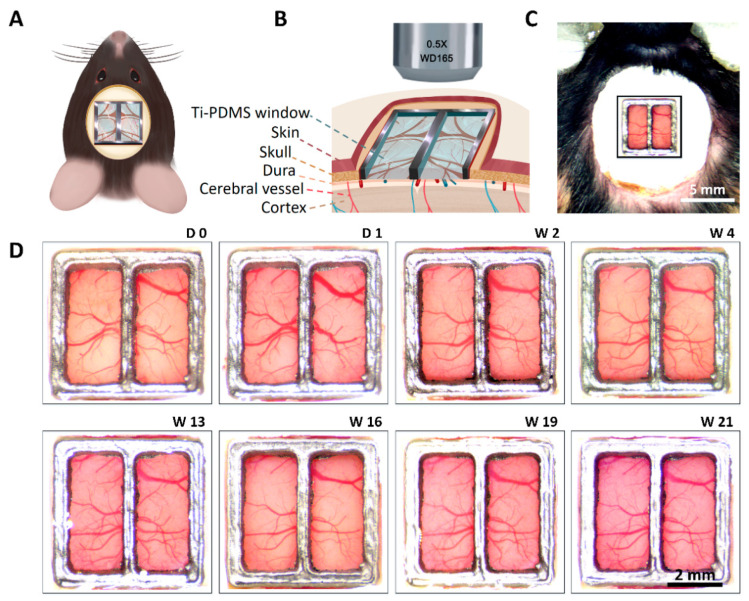
Implantation and functional evaluation of chronic Ti-PDMS cranial window. (**A**) Schematic diagram of a rodent with Ti-PDMS cranial window. (**B**) Schematic cross-section of a rodent with Ti-PDMS cranial window. (**C**) A typical photograph of one C57BL/6 mouse with Ti-PDMS cranial window. (**D**) Cortical images of the Ti-PDMS cranial window at D 0, D 1, W 2, W 4, W 13, W 16, W 19 and W 21 post-implantation.

Figure 6A,B were schematic diagrams for the *in vivo* implantation of Ti-PDMS cranial window. Ti-PDMS cranial windows were implanted in 22 mice. Among them, some of the cranial window implant surgery failed due to un-absorbable cerebral hemorrhage or opacity. These experiments were terminated within 2 weeks after surgery. The success rate of craniotomy surgery was 72.73% (n = 16/22 mice). The duration of implantation was from 2 to 21 weeks, with an average duration of 8.6 weeks.

Figure 6C showed a Ti-PDMS implantation at the center of its cranium through a craniotomy (see Materials and Methods, Figure S5 in Supplementary Materials for details). For successful implantation, cortical blood vessels and tissues kept healthy conditions, PDMS windows kept clear and transparent, no breaking or crack was observed in PDMS windows (0.35–0.5 mm thickness), and no obvious infection or discomfort happened. Even 21 weeks after implantation, PDMS windows kept well. Cortical images of the chronic Ti-PDMS cranial window were captured at D 0, D 1, W 2, W 4, W 13, W 16, W 19 and W 21 post-implantation (Figure 6D). Figure S8 in Supplementary Materials showed cortical images from another mouse at 0, 4, 8, 11, 15, 18, 26 and 63 days post-implantation. The living conditions of mice after 2-week implantation were shown in Video S4 and S5 in Supplementary Materials.

These results prove that the Ti-PDMS cranial windows have excellent bio-compatibility, and have sufficient mechanical strength for long-period *in vivo* neuroimaging and observation.

### 3.6. Multi-Site Injection and In Vivo Two-Photon Imaging through Ti-PDMS Cranial Window

In order to explore the brain function and effectively treat brain diseases, it is usually necessary to directly access the brain tissue *in vivo*. Ti-PDMS cranial window provides a path for combining multiple techniques to record and/or interfere with brain function. The soft and hydrophobic PDMS window allows intracortical injection of dyes or drugs without liquid leakage. The glass micropipette easily penetrated from any position. In terms of imaging, the spatial resolution and imaging depths of Ti-PDMS cranial windows were equivalent to those of glass cranial windows [32,55]. 

**Figure 7:**
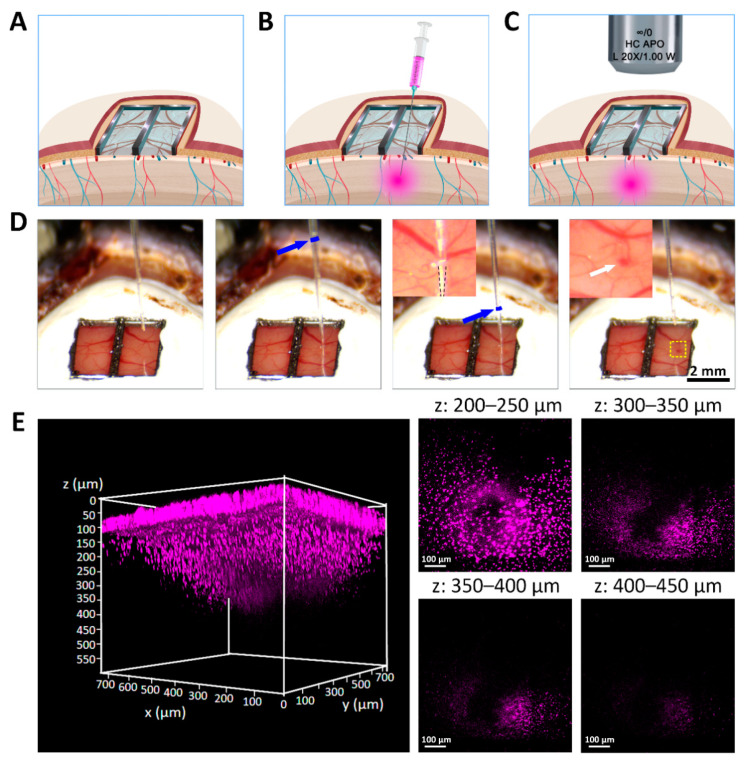
Dye injection of Hoechst 33258 and *in vivo* two-photon imaging through Ti-PDMS cranial window. (**A**–**C**) Schematic diagram of single site injection and imaging through Ti-PDMS cranial window. (**D**) Sequential images of the glass pipette placement and removal performed in 10 weeks post-implantation. A glass pipette was inserted into the brain tissue directly through the PDMS cover to avoid major vasculature. Hoechst 33258 (10 µg mL^−1^, 0.2 µL, 0.1 µL min^−1^) was injected into the brain. The blue arrow in the second and third images marked the liquid level. The black dashed lines inserted in the third image outlined the tip of the glass pipette. The yellow dashed line in the fourth image marked the injection site. The image inserted in the fourth image showed the enlarged view of the injection site, which was marked by the white arrow. (**E**) Two-photon imaging for Hoechst 33258 was possible up to 450 µm in z-depth.

The schematic diagram Figure 7A–C showed the process of injecting fluorescent dyes into the mouse brain and performing two-photon imaging *in vivo*. Hoechst 33258 fluorescent dye (0.2 µL) was injected into the cortex through the Ti-PDMS cranial window with a glass micropipette and nanoliter-injector. In this experiment, the injection site was selected to avoid large blood vessels. Figure 7D and Video S6 in Supplementary Materials showed the process of injecting fluorescent dye into the brain at 70 days post-implantation. The tip of the micropipette was located approximately 0.7 mm below the cortex surface. As shown in the fourth image in Figure 7D, no obvious bleeding or liquid leakage was founded after slowly pulling out the micropipette at a speed of 0.5 mm min^−1^. This was due to the self-sealing and high hydrophobic properties of the PDMS window [32,73]. The measurement results of the contact angle of the PDMS film were shown in Figure S4 in Supplementary Materials.

At 20 min after the injection, longitudinal two-photon imaging of Hoechst 33258 (magenta) were observed within the depth of 600 µm. The insertion position can be confirmed through the confocal image on PDMS cover, as indicated by the white arrow (Figure S6A and Video S7, in Supplementary Materials). The fluorescent signal at depths of 200–250 µm, 300–350 µm, 350–400 µm and 400–450 µm were clearly observed (Figure 7E and Video S8, in Supplementary Materials). No fluorescent signal was seen in non-injection site (Figures S6B and S9, Video S9 and S10, in Supplementary Materials). Further, the results of *in vitro* experiments showed that PDMS (0.3 mm thick) remained self-sealing without any fluid leakage after 15 injections at one site with a commercial 1 mL syringe (Figure S7, Video S1, in Supplementary Materials). If injected too many times or the object being inserted is too large (greater than 20 gauge, 0.51 mm), the PDMS may rupture, but this was easily remedied by applying fast-setting cyanoacrylate glue over the rupture [32].

In addition, DiI and Cholera toxin subunit B (recombinant), Alexa Fluor^®^ 488 conjugate (CTB488) were injected into three spots through PDMS. There was no obvious bleeding and fluid leakage after the micropipette was removed. Two days after injection, the distribution of fluorescent at three sites was visualized by confocal imaging, as shown in Figure S10 and Video S11 in Supplementary Materials. These results strongly indicate that our artificial cranial window combined with Ti alloy frame and PDMS film provides a convenient way for multi-site injections to avoid cerebral blood. This is of great significance to study brain function and treat brain disorders.

In this paper, we prove that a hybrid titanium-softmaterial, high-strength, transparent cranial window is suitable for transcranial injection and neuroimaging. Ti-PDMS cranial window will help to reduce the number of small animals for two main reasons. First, this Ti-PDMS device offers an alternative choice for *in vivo* optical experiments, ultrasonic treatment and electrophysiology recording. It is possible to explore the brain with multiple-level methods in the same animal. In addition, as for the “self-sealing” property, PDMS allows not only multiple injections (at least 15 times) in the same brain area, but also multi-site injections in different area. It is very useful for chronic functional brain research and reducing the number of small animals.

There are also some limitations in our study. Whether Ti-PDMS cranial windows are suitable for functional imaging, such as calcium imaging, requires further experimental demonstration. We are designing functional experiment to explore the new applications of Ti-PDMS cranial windows. In addition, the size of Ti-PDMS device (5 mm × 5 mm × 0.35 mm) is relatively small in this experiment. In the future, we will design customize complex detachable and replaceable cranial window devices that can cover cortical regions for large animals. The complex cranial window device may integrate multiple modules such as micro-processing system, micro-microscope, speckle metamaterial-assisted illumination nanoscopy, brain-computer interface and its chip, microfluidic channel, ultrasound window and light window, which will bring breakthroughs in the research of brain mechanism and the treatment of brain diseases.

## 4. Conclusions

In summary, a series of high-strength hybrid Ti-PDMS artificial cranial windows were developed for living animals, which allowed a high proportion of transparent area for *in vivo* observation and two-photon analysis, and for trans-window injection. Laser scanning and 3D printing techniques were used to match the hybrid cranial window to different skull morphology. After mechanical polishing treatment for the surface, the Ti frames were flat and would not cause tissue damage or bleeding. A multi-cycle degassing pouring process ensured a good combination of PDMS and Ti frame without bubbles. Furthermore, Ti-PDMS cranial windows had a high fracture strength matching human skull bone. Meanwhile, the average light transmittance of the PDMS window used in our devices was better than that of commercial #1 glass coverslip for bio-experiments, and refractive index was close to biological tissue. Ti-PDMS cranial windows showed excellent bio-compatibility during 21-week implantation in mice, and satisfied the requirement for short- and long-term investigations. Additionally, Dye injection showed that the PDMS window had a “self-sealing” to keep liquid from leaking out. Two-photon imaging for brain tissues could be achieved up to 450 µm in z-depth. This Ti-PDMS device offers an alternative and universal choice for a variety of *in vivo* drug delivery, optical experiments, ultrasonic treatment, as well as multi-functional brain-computer-interfaces.

## Data Availability

The data presented in this study are available on request from the corresponding author.

## Supplementary Materials

The following supporting information can be downloaded at: https://www.mdpi.com/article/10.3390/bios12020129/s1, Figure S1: Design patterns of Ti frames with different shapes and sizes. Figure S2: 3D reconstruction model of rat skull specimen. Figure S3: AFM images of the polished inner surface of Ti frame (A) and Ti-PDMS cranial window sample (B). Figure S4: Static contact angle images of PDMS surface. Figure S5: Overall surgical procedures for the implantation of the Ti-PDMS cranial window in a mouse. Figure S6: Two-photon images of Hoechst 33258. Figure S7: Sequential images of pre-injection, first, fifth, tenth, fifteenth injection, and post-injection. Figure S8: Cortical images of the Ti-PDMS cranial window at 0, 4, 8, 11, 15, 18, 26, and 63 days post-implantation. Figure S9: Two-photon images of a site on the non-injection side within a depth of 600 µm. Figure S10: The confocal imaging for the whole Ti-PDMS cranial window and three injection sites of a mouse. Table S1: Chemical composition of Ti-6Al-4V alloy. Table S2: Weight of Ti-PDMS cranial window for different animals. Video S1: Representative video of 15 injections performed through a commercial 1 mL syringe penetrating the PDMS membrane. Video S2: Representative two-photon imaging of the fluorescent signal through PDMS. Video S3: Representative two-photon imaging of the fluorescent signal through a glass coverslip. Video S4: Representative videos of mouse behavior at 2 weeks post Ti-PDMS cranial window implantation in light. Video S5: Representative videos of mouse behavior at 2 weeks post Ti-PDMS cranial window implantation in dark. Video S6: Representative video of directly injected Hoechst 33258 fluorescent dye into the cortex through Ti-PDMS cranial window with a glass micropipette. Video S7: Representative two-photon imaging of Hoechst 33258 (magenta) in sites of injection side in the brain of the mouse implanted with Ti-PDMS cranial window. Video S8: Representative two-photon imaging for Hoechst 33258 through Ti-PDMS cranial window in injection site. Video S9: Representative two-photon imaging in sites of non-injection side in the brain of the mouse implanted with Ti-PDMS cranial window. Video S10: Representative two-photon imaging through Ti-PDMS cranial window in a site on the non-injection side. Video S11: Representative confocal imaging of the whole Ti-PDMS cranial window and three injection sites.
