# Supplementary material for: A Hybrid Titanium-Softmaterial, High-Strength, Transparent Cranial Window for Transcranial Injection and Neuroimaging
Source: Biosensors (Basel). 2022 Feb 18;12(2):129. doi: 10.3390/bios12020129 (PMC8870569; doi:10.3390/bios12020129)
Supplement: Supplementary file 1 [file biosensors-12-00129-s001.zip › biosensors-1546879-supplementary.pdf]

biosensors

MDPI

Article

A Hybrid Titanium-Softmaterial, High-Strength, Transparent Cranial Window for Transcranial Injection and Neuroimaging

Nana Yang [1], Fengyu Liu [2,3,*], Xinyue Zhang [4,5], Chenni Chen [2,3], Zhiyuan Xia [6], Su Fu [2,3], Jiaxin Wang [2,3], Jingjing Xu [1,7], Shuang Cui [2,3], Yong Zhang [2,3], Ming Yi [2,3], You Wan [2,3], Qing Li [4,5], Shengyong Xu [1,*]

1 Key Laboratory for the Physics & Chemistry of Nanodevices, Department of Electronics, Peking University, 100871 Beijing, P. R. China; yangnana@pku.edu.cn (N.Y.); xujj@sdu.edu.cn (J.X.)
2 Neuroscience Research Institute and Department of Neurobiology, School of Basic Medical Sciences, Peking University, 100191 Beijing, P. R. China; ccn@pku.edu.cn (C.C.); sufu@bjmu.edu.cn (S.F.); Jiaxinw@bjmu.edu.cn (J.W.); cshuang@bjmu.edu.cn (S.C.); yongzhang@hsc.pku.edu.cn (Y.Z.); mingyi@bjmu.edu.cn (M.Y.); ywan@hsc.pku.edu.cn (Y.W.)
3 Key Laboratory for Neuroscience, Ministry of Education/National Health Commission, Peking University, 100191 Beijing, P. R. China
4 Center of Digital Dentistry, Peking University School and Hospital of Stomatology, 100081 Beijing, P. R. China; zhangxinyue@pkuss.bjmu.edu.cn (X.Z.); qingli@bjmu.edu.cn (Q.L.)
5 National Engineering Laboratory for Digital and Material Technology of Stomatology, 100081 Beijing, P. R. China
6 Department of Material Science and Engineering, College of Engineering, Peking University, 100871 Beijing, P. R. China; xiazy@pku.edu.cn (Z.X.)
7 School of Microelectronics, Shandong University, 250100 Jinan, P. R. China
* Correspondence: liufyu@bjmu.edu.cn (F.L.); xusy@pku.edu.cn (S.X.)

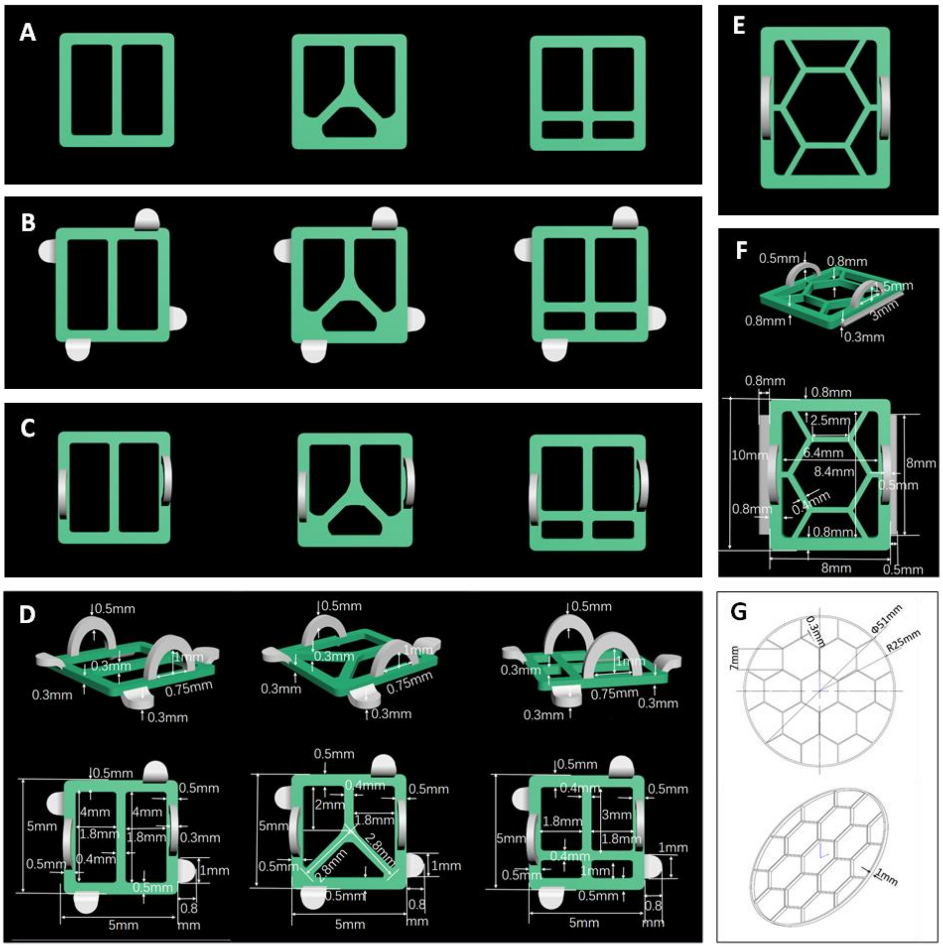

Figure S1. Design patterns of Ti frames with different shapes and sizes. (A–D) Design patterns of square Ti frames suitable for mice with grids defining varied inner opening shapes (A), with small locking parts (B), with small clamping parts (C), with both locking and clamping parts (D). (E,F) Design patterns of rectangular Ti frames suitable for rats with clamping parts (E), with both locking and clamping parts (F). (G) Design patterns of circular Ti frame with hexagonal meshes suitable for large animals.

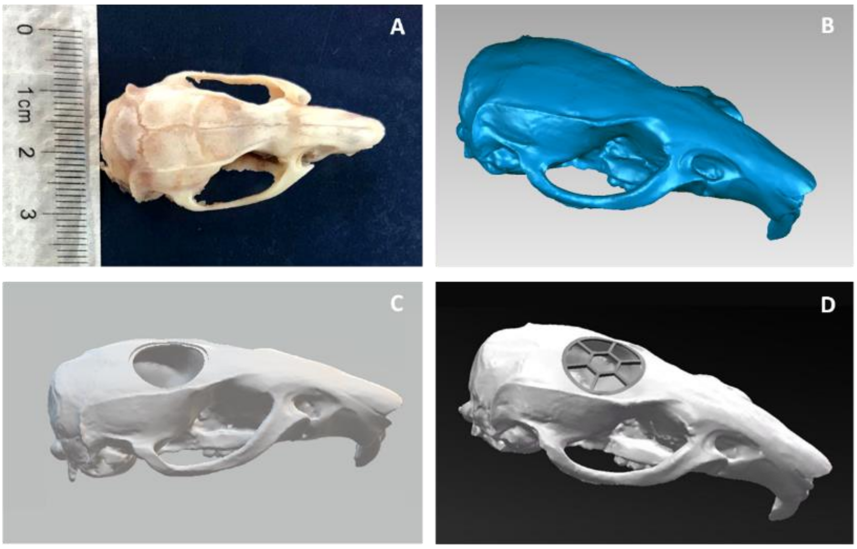

Figure S2. 3D reconstruction model of rat skull specimen. (A) A typical skull specimen of rat. (B) 3D morphology of rat skull specimen. (C) 3D model of rat skull specimen with a circular location modified by 3D Sprint software. (D) 3D model of rat skull specimen with a curved Ti frame modified by 3D Sprint software.

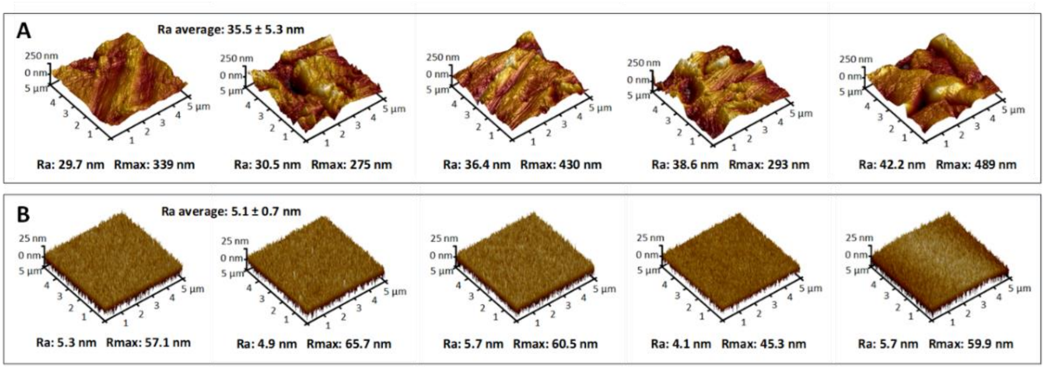

Figure S3. AFM images of the polished inner surface of Ti frame (A) and Ti-PDMS cranial window sample (B).

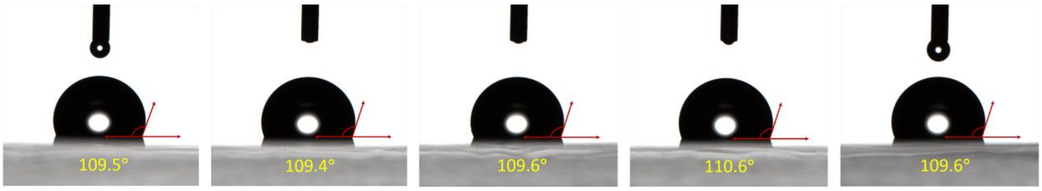

Figure S4. Static contact angle images of PDMS surface.

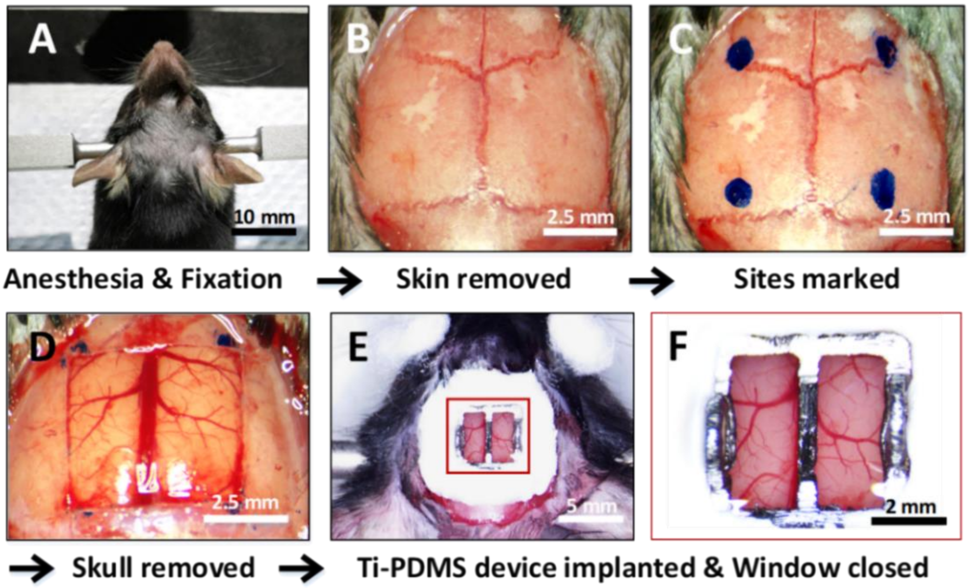

Figure S5. Overall surgical procedures for the implantation of the Ti-PDMS cranial window in a mouse. (A) The mouse was anesthetized and firmly fixed in the stereotaxic instrument. (B) The scalp of the mouse was carefully removed to expose the skull. (C) Sites of the craniotomy was marked with a marker. (D) The scalp of the mouse was carefully removed. (E) The Ti-PDMS cranial window was placed over the exposed brain tissue area and fixed with dental resin. (F) An enlarged view of the Ti-PDMS cranial window marked by the red box in (E).

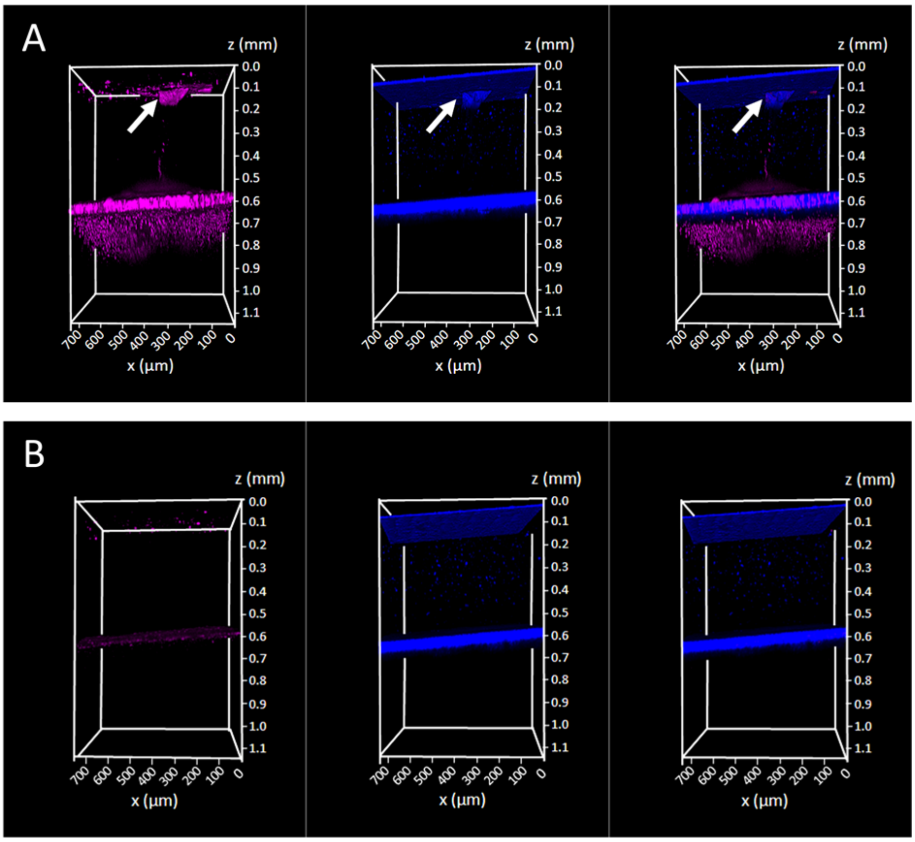

Figure S6. Two-photon images of Hoechst 33258. (A) Two-photon images of Hoechst 33258 (magenta) in the injection site on the mouse implanted with Ti-PDMS cranial window. The white arrow marked the insertion position on PDMS. The reflected light (blue) of PDMS surface and brain surface were imaged. (B) Two-photon images of a site on the non-injection side.

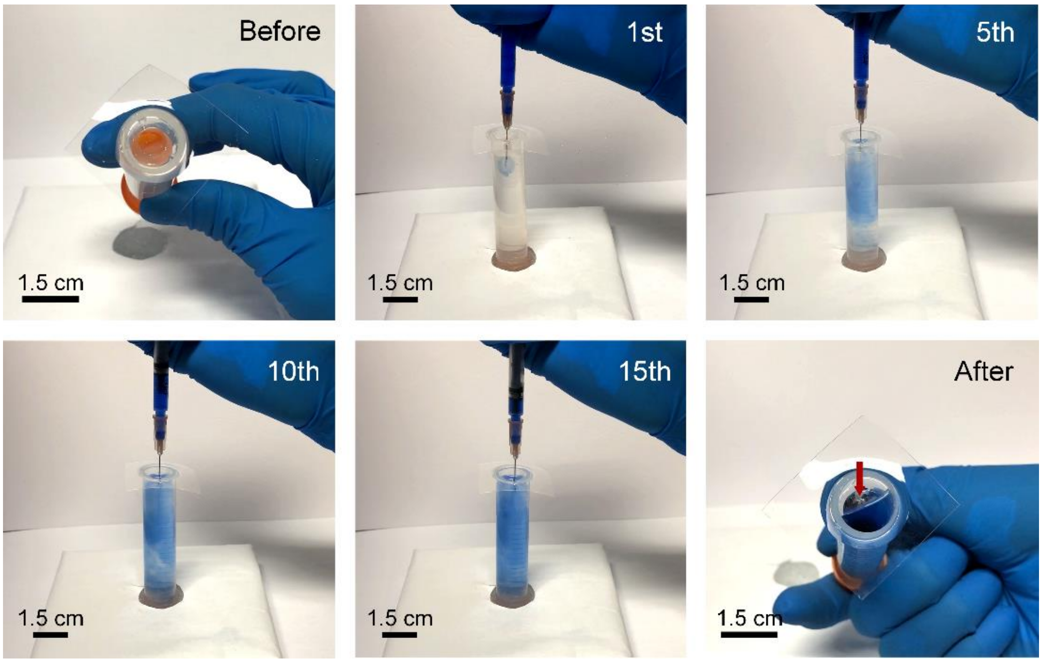

Figure S7. Sequential images of pre-injection, first, fifth, tenth, fifteenth injection, and post-injection. The red arrow in the last image marked the injection site.

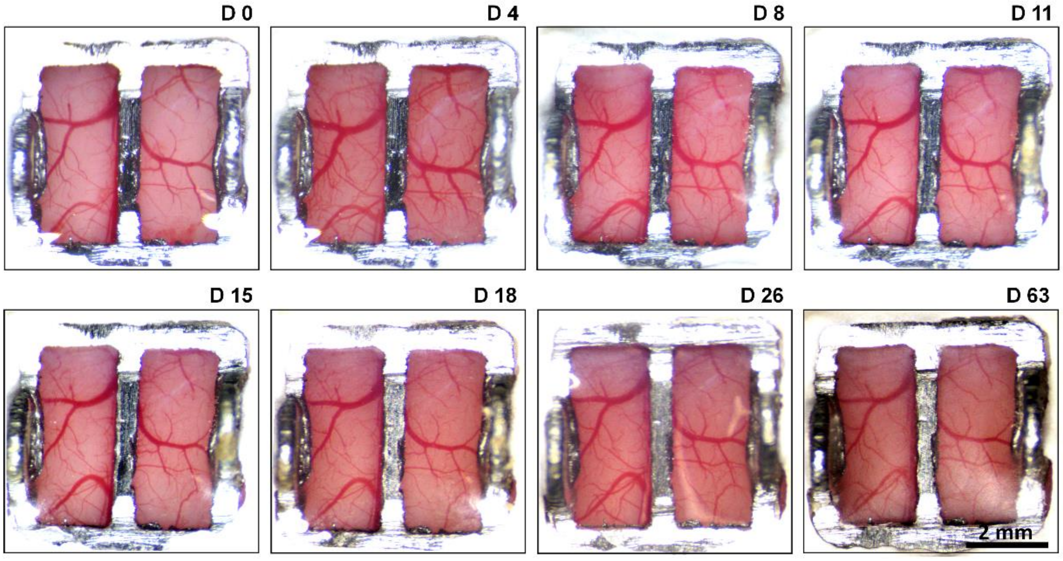

Figure S8. Cortical images of the Ti-PDMS cranial window at 0, 4, 8, 11, 15, 18, 26, and 63 days post-implantation.

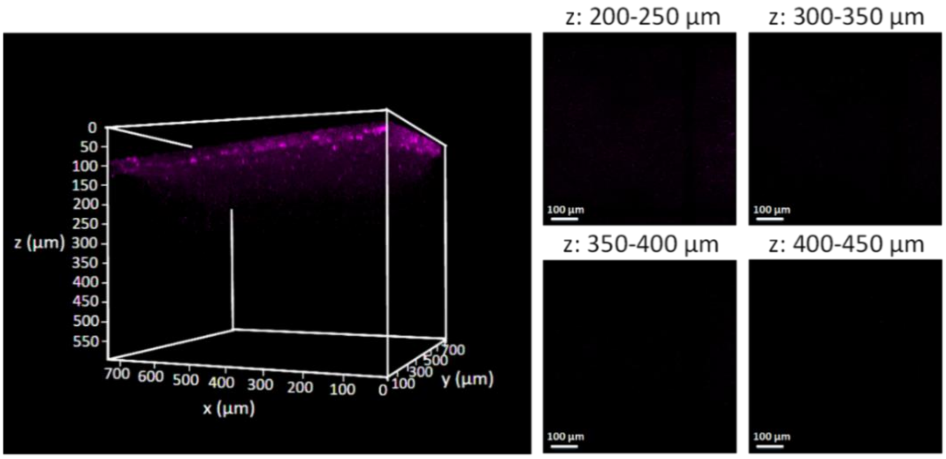

Figure S9. Two-photon images of a site on the non-injection side within a depth of 600 μm.

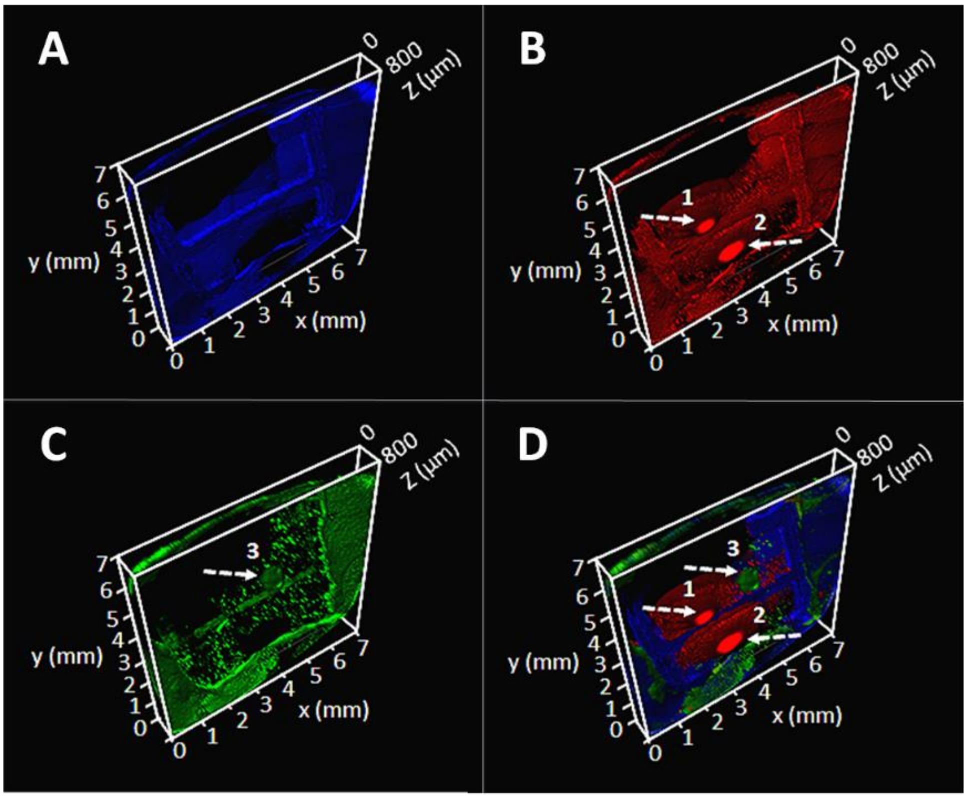

Figure S10. The confocal imaging for the whole Ti-PDMS cranial window and three injection sites of a mouse. (A) A confocal image of the whole Ti-PDMS cranial window. (B) A confocal image of the first and second injection sites, the red DiI dye was injected into these two sites, which were marked by white dotted arrow. (C) A confocal image of the third site, the green CTB488 dye was injected into this site, which was marked by white dotted arrow. (D) The merged image from A to C.

Table S1. Chemical composition of Ti-6Al-4V alloy (mass fraction, %)

Element	Al	V	Fe	O	C	N	H	Y	Ti
**Mass Fraction (%)**	5.50–6.75	3.50–4.50	≤0.30	≤0.20	≤0.08	≤0.05	≤0.015	≤0.005	Balance

Table S2. Weight of Ti-PDMS cranial window for different animals

Sample Number	Weight of Ti-PDMS Cranial Window for Mouse (g)	Weight of Ti-PDMS Cranial Window for Rat (g)	Weight of Ti-PDMS Cranial Window for Large Animal (g)
**Sample #1**	0.0481	0.20	5.82
**Sample #2**	0.0483	0.20	6.70
**Sample #3**	0.0404	0.21	7.06
**Sample #4**	0.0302	0.19	6.86
**Sample #5**	0.0492	0.18	5.91
**Sample #6**	0.0494	0.21	7.23
**Sample #7**	0.0471	0.22	6.36
**Sample #8**	0.0436	0.23	6.06
**Sample #9**	0.0382	0.21	7.15
**Sample #10**	0.0353	0.24	6.28
**Mean**	**0.04298**	**0.21**	**6.54**
**Standard Deviation**	**0.00669**	**0.02**	**0.53**

Supplementary Videos

Video S1, related to Figure S7. Representative video of 15 injections performed through a commercial 1 mL syringe penetrating the PDMS membrane.

Video S2 and Video S3, related to Figure 5E and **Figure 5F,** respectively. Representative two-photon imaging of the fluorescent signal through PDMS (**Video S2**) and a glass coverslip (**Video S3**). Note that the resolution of the Videos was reduced after conversion from Adobe Premiere Pro software to Video format mp4.

Video S4 and **Video S5,** Representative videos of mouse behavior at 2 weeks post Ti-PDMS cranial window implantation in light (**Video S4**) and dark (**Video S5**). A single animal was housed in its own home cage after cranial window implantation surgery. The animal exhibited normal grooming, drinking, feeding, and sleeping behavior.

Video S6, related to Figure 7D. Representative video of directly injected Hoechst 33258 fluorescent dye into the cortex through Ti-PDMS cranial window with a glass micropipette. The micropipette was removed without creating any bleeding or CSF leakage.

Video S7, related to Figure S6A, Representative two-photon imaging of Hoechst 33258 (magenta) in sites of injection side in the brain of the mouse implanted with Ti-PDMS cranial window.

Video S8, related to Figure 7E. Representative two-photon imaging for Hoechst 33258 through Ti-PDMS cranial window in injection site.

Video S9, related to Figure S6B, Representative two-photon imaging of Hoechst 33258 in sites of non-injection side in the brain of the mouse implanted with Ti-PDMS cranial window.

Video S10, related to Figure S9. Representative two-photon imaging through Ti-PDMS cranial window in a site on the non-injection side.

Video S11, related to Figure S10. Representative confocal imaging of the whole Ti-PDMS cranial window and three injection sites.
